# Metabolomics Analysis Reveals Altered Metabolic Pathways and Response to Doxorubicin in Drug-Resistant Triple-Negative Breast Cancer Cells

**DOI:** 10.3390/metabo13070865

**Published:** 2023-07-20

**Authors:** Blake R. Rushing, Sabrina Molina, Susan Sumner

**Affiliations:** 1 Department of Nutrition, University of North Carolina at Chapel Hill, Chapel Hill, NC 27599, USA; 2 Nutrition Research Institute, University of North Carolina at Chapel Hill, Kannapolis, NC 28081, USA

**Keywords:** metabolomics, triple-negative breast cancer, drug resistance, doxorubicin, chemotherapy

## Abstract

This study aimed to investigate metabolic changes following the acquisition of resistance to doxorubicin in the triple-negative breast cancer (TNBC) cell line MDA-MB-231. Two drug-resistant cell lines, DOX-RES-50 and DOX-RES-100, were generated by treating MDA-MB-231 cells with doxorubicin for 24 h and allowing them to recover for six weeks. Both drug-resistant cell lines demonstrated an increase in doxorubicin IC_50_ values, indicating acquired drug resistance. Metabolomics analysis showed clear separation between the parental MDA-MB-231 cell line and the drug-resistant cell lines. Pathway analysis revealed that arginine and proline metabolism, glutathione metabolism, and beta-alanine metabolism were significantly perturbed in the drug-resistant cell lines compared to the parental cell line. After matching signals to an in-house library of reference standards, significant decreases in short- and medium-chain acylcarnitines and significant increases in long-chain acylcarnitines, 5-oxoproline, and 7-ketodeoxycholic acid were observed in the resistant cell lines as compared to the parental MDA-MB-231 cell line. In addition to baseline metabolic differences, we also investigated differences in metabolic responses in resistant cell lines upon a second exposure at multiple concentrations. Results indicate that whereas the parental MDA-MB-231 cell line had many metabolites that responded to doxorubicin in a dose-dependent manner, the two resistant cell lines lost a dose-dependent response for the majority of these metabolites. The study’s findings provide insight into how metabolism is altered during the acquisition of resistance in TNBC cells and how the metabolic response to doxorubicin changes upon repeated treatment. This information can potentially identify novel targets to prevent or reverse multi-drug resistance in TNBC, and also demonstrate the usefulness of metabolomics technology in identifying new mechanisms of drug resistance in cancer and potential drug targets.

## 1. Introduction

In the United States, breast cancer is the most diagnosed cancer for women (excluding skin cancers) and is the second leading cause of cancer deaths in women [1]. One subtype of breast cancers—triple-negative breast cancer (TNBC)—accounts for approximately 10–15% of all breast cancer cases and carries the worst prognosis of hormone-receptor-positive breast cancers [2]. While there are several treatment options available for TNBC, drug resistance remains a significant challenge in managing this disease. Chemotherapy remains the cornerstone of TNBC treatment and TNBCs can be inherently resistant to drug therapy (intrinsic resistance), or they can become resistant following an initial exposure to the drug (acquired resistance) [3]. In general, the initial response of TNBCs to chemotherapy is favorable; however, the majority of patients will relapse with disease that is drug-resistant and prone to metastasis, which eventually becomes fatal [4]. One of the major causes of drug resistance in breast cancer is the genetic diversity of cancer cells. Cancer cells can mutate and adapt to their environment, which can make them resistant to chemotherapies [5]. Additionally, cancer cells can develop mechanisms to bypass the drugs used to treat them, such as developing alternative signaling pathways that allow them to grow and survive. Cancer stem cells, a cellular subpopulation in tumors with self-renewing capabilities, are also generally more drug-resistant as compared to more differentiated cancer cells, and allow for the reestablishment of tumors after drug treatment [6]. More research is needed to better understand the mechanisms underlying drug resistance in TNBC and develop more effective therapies to overcome it.

Doxorubicin is a commonly used chemotherapeutic drug for the treatment of TNBC [7]. The mechanisms of doxorubicin resistance in breast cancer are complex and multifactorial, and developing strategies to overcome them is essential for improving the effectiveness of doxorubicin in breast cancer treatment. One of the primary mechanisms of doxorubicin resistance in breast cancer is decreased drug uptake. Doxorubicin enters cancer cells through specific transporters, and decreased expression or activity of these transporters can lead to reduced drug uptake and lower drug efficacy [7]. Another important mechanism of doxorubicin resistance is increased drug efflux. Cancer cells can actively pump doxorubicin out of the cell through efflux pumps, such as P-glycoprotein (P-gp) and ATP-binding cassette (ABC) transporters, leading to lower intracellular drug concentrations and reduced efficacy [8,9]. Altered drug metabolism is also a potential mechanism of doxorubicin resistance in breast cancer. Doxorubicin is metabolized in the liver by enzymes such as cytochrome P450 (CYP) 3A4, and genetic variations in these enzymes can affect the metabolism and clearance of doxorubicin, leading to variable drug responses [10]. Additionally, increased DNA repair capacity can make cancer cells more resistant to doxorubicin. Doxorubicin acts by damaging the DNA of cancer cells, but cancer cells with increased DNA repair capacity can more effectively repair this damage, reducing the effectiveness of the drug [11]. Changes in apoptotic pathways can also contribute to doxorubicin resistance in breast cancer, with changes in apoptotic pathways, such as increased expression of anti-apoptotic proteins, increasing cancer cell resistance to the drug [11]. Finally, changes in the tumor microenvironment can also affect the drug response. Factors such as hypoxia, acidosis, and inflammation can lead to doxorubicin resistance in breast cancer [12].

An understudied factor in the development of doxorubicin resistance is cancer cell metabolism. Metabolic reprogramming is a hallmark of cancer and has been shown to play a critical role in drug resistance [13]. Cancer cells undergo significant metabolic changes to support their rapid proliferation, survival, and adaptation to different environments, and these metabolic changes can lead to altered drug responses and drug resistance [14,15]. In addition to modification of the metabolic pathway of drugs themselves, modification of signaling pathways that regulate endogenous cancer cell metabolism, such as the PI3K/Akt/mTOR pathway, have been shown to play a role in drug resistance. Activation of these pathways can promote cell survival, reduce apoptosis, and enhance DNA repair, all of which contribute to drug resistance [11]. Much still remains unknown about the metabolic adaptations that TNBC cells undergo to acquire resistance to doxorubicin treatment. A better understanding of these processes can lead to the identification of biomarkers for the prediction of treatment response, or the identification of sensitization targets to improve TNBC therapy response.

**Figure 1:**
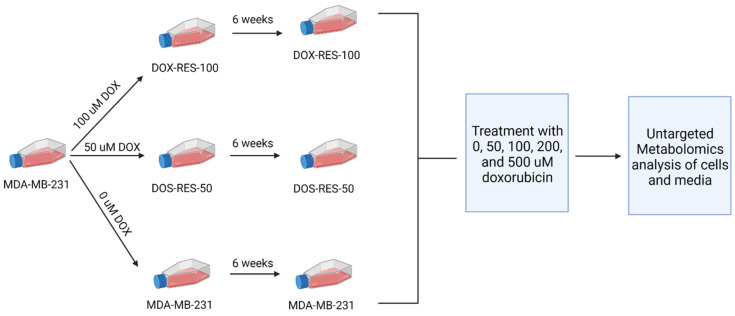
Schematic of experimental design. Parental MDA-MB-231 cells were treated with 0, 50, or 100 μM doxorubicin for 24 h and then allowed to recover over a six-week period. Each cell line was then treated with a dose range of doxorubicin for 24 h. Cells and media samples were collected and extracted for untargeted metabolomics analysis. Analysis of intracellular (cells) and extracellular (media) metabolites was performed separately, as described below.

Metabolomics technologies can be used to identify new mechanisms of drug resistance in cancer by analyzing the metabolic profile of cancer cells before and after treatment with drugs [16]. By analyzing the metabolite profiles of cancer cells before and after drug treatment, metabolomics can identify altered metabolic pathways and metabolic biomarkers of drug resistance or response, and reveal new drug targets and drug combinations to overcome resistance. In the current investigation, we present the intracellular and extracellular metabolic changes that occur following acquired resistance in the TNBC cell line MDA-MB-231. We also present the differences in the metabolic response to doxorubicin treatment in the parental MDA-MB-231 cell line and our drug-resistant cells (Figure 1). We present first the changes in the intracellular (cell) samples and then the extracellular (media) samples. This information provides insight into how metabolism is altered during the acquisition of resistance in TNBC cells as well as how the metabolic response to doxorubicin changes upon repeated treatment, potentially identifying novel targets to prevent or reverse multi-drug resistance (MDR) in TNBC.

## 2. Results

**Figure 2:**
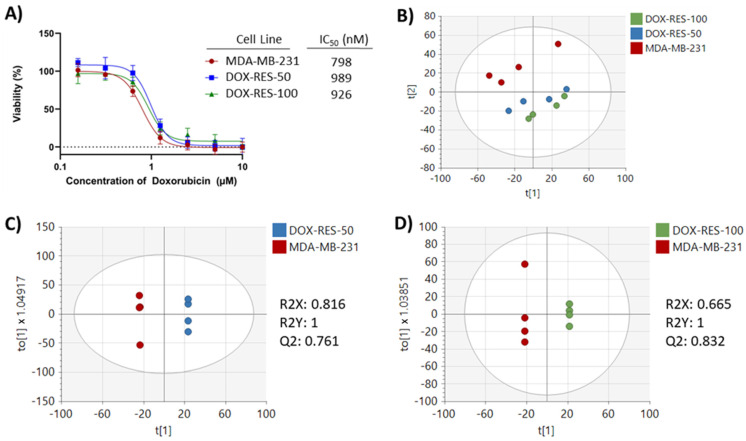
(**A**) Viability dose–response curves of each cell line following doxorubicin treatment and their calculated IC_50_ values. (**B**) PCA of intracellular metabolomics data of MDA-MB-231, DOX-RES-50, and DOX-RES-100 treated with 0 μM doxorubicin. (**C**) OPLS-DA of intracellular metabolomics data of MDA-MB-231 and DOX-RES-50. (**D**) OPLS-DA of intracellular metabolomics data of MDA-MB-231 and DOX-RES-100.

Two drug-resistant cell lines were generated by treating MDA-MB-231 cells with doxorubicin for 24 h and then allowing the cells to recover over the course of six weeks. One drug-resistant cell line, DOX-RES-50, was produced by treating MDA-MB-231 cells with 50 µM doxorubicin for 24 h, while another drug-resistant cell line, DOX-RES-100, was produced by treating MDA-MB-231 cells with 100 µM doxorubicin for 24 h. After the six-week recovery period, both cell lines along with the parental MDA-MB-231 were assessed for doxorubicin sensitivity using a viability assay. Both the DOX-RES-50 and the DOX-RES-100 cell lines showed an increase in doxorubicin IC_50_ values, demonstrating the acquisition of drug resistance (Figure 2A). In order to understand the metabolic differences between the drug-resistant and parental cells, metabolomics analysis was performed on each cell line.

After the preprocessing of metabolomics data, 4063 features remained for analysis. Principal component analysis (PCA) showed clear separation between the parental MDA-MB-231 cell line and the drug-resistant cell lines. Additionally, the DOX-RES-50 and DOX-RES-100 cell lines showed noticeable overlap in the PCA plot, indicating similarities in metabolic profiles (Figure 2B). Pairwise orthogonal partial least squares discriminant analysis (OPLS-DA) was performed to further verify differences between the MDA-MB-231 cells and each drug-resistant cell line (R2Y, Q2 > 0.5) and to assign variable importance to projection (VIP) values to each peak to identify signals with high discrimination potential (Figure 2C,D). Fold changes and *p*-values were also calculated for each peak to further understand signals differentiating each pairwise comparison (Supplemental Table S1). Significant differences were found in 373 and 572 peaks (*p* < 0.05) in DOX-RES-50 and DOX-RES-100 cells, respectively, compared to the parental MDA-MB-231 cell line.

**Figure 3:**
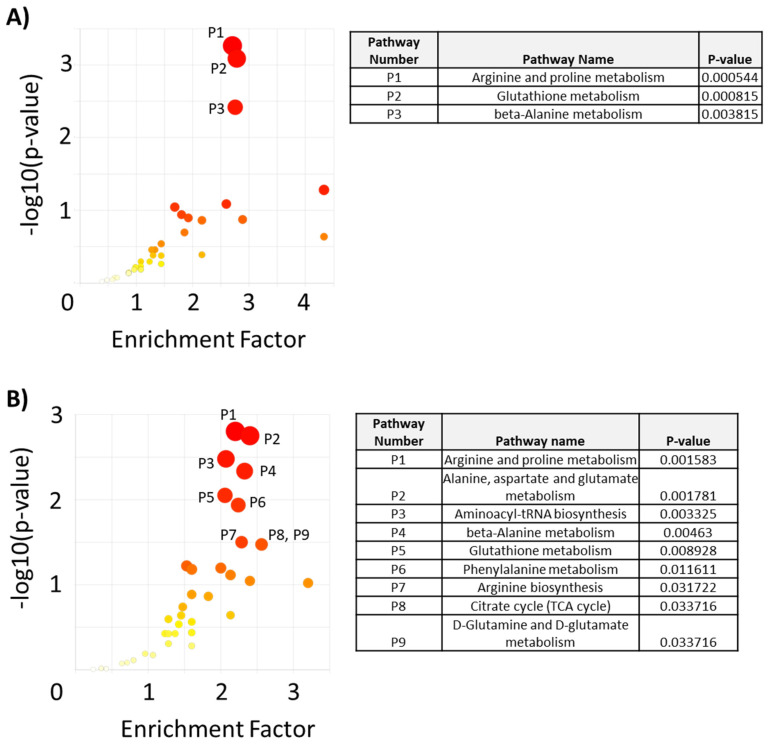
Pathway analysis of intracellular metabolomics data showing differentiating metabolic pathways between (**A**) MDA-MB-231 and DOX-RES-50 and (**B**) MDA-MB-231 and DOX-RES-100. Pathways with a *p* < 0.05 are annotated. Darker red colors signify a higher value on the y-axis.

Pathway analysis was performed using the mummichog algorithm of MetaboAnalyst 5.0 using all metabolomics peaks and their corresponding *p*-values for the MDA-MB-231 versus DOX-RES-50 and the MDA-MB-231 versus DOX-RES-100 comparisons. Results revealed that arginine and proline metabolism, glutathione metabolism, and beta-alanine metabolism were significantly perturbed between the MDA-MB-231 and DOX-RES-50 cells (Figure 3A). These three pathways were also significantly perturbed in the DOX-RES-100 cells, along with several additional pathways (Figure 3B). Notably, arginine and proline metabolism were the top pathway by *p*-value for both drug-resistant cell lines.

**Figure 4:**
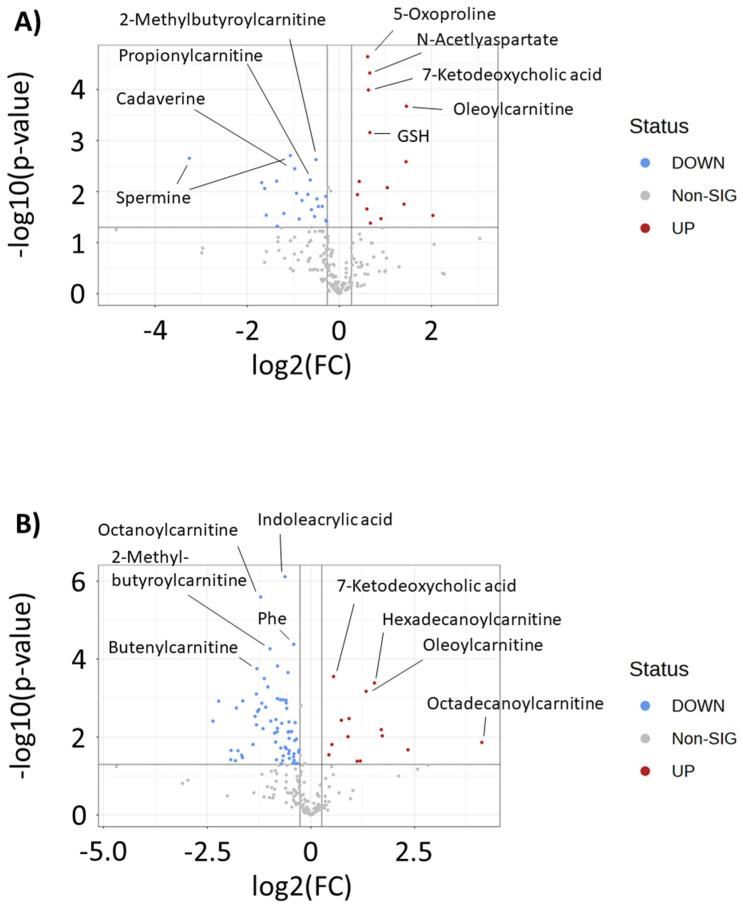
Volcano plots of intracellular in-house matched metabolites that differentiate (**A**) MDA-MB-231 and DOX-RES-50 and (**B**) MDA-MB-231 and DOX-RES-100. Metabolites are highlighted that have a *p* < 0.05 and an absolute fold change ≥1.2. Metabolites in red are increased in doxorubicin-resistant cell line samples and metabolites in blue are decreased in doxorubicin-resistant cell line samples.

To further understand metabolites that differentiate drug-resistant cells from the parental MDA-MB-231 cell line, we matched metabolomics signals to in-house libraries and public databases. This resulted in 235 in-house library matches and 2786 public database matches. Each match, along with an ontology level to signify the evidence basis underlying each match, is given in Supplemental Table S1. To aid in confirming the significant pathways listed in Figure 3, we identified creatine, spermidine, spermine, and 4-acetamidobutanoic acid as decreased, and 5-oxoproline and reduced glutathione as significantly increased, in one or both drug-resistant cell lines using our in-house library. Additionally, volcano plot analysis was performed to reveal the in-house matched metabolites that were most significantly increased or decreased in each drug-resistant cell line. Analysis revealed significant decreases in short- and medium-chain acylcarnitines (2-methylbutyroylcarnitine, propionylcarnitine, butyroylcarnitine, butenylcarnitine) and increases in long-chain acylcarnitines (oleoylcarnitine, hexadecanoylcarnitine, octadecanoylcarnitine). Notably, 5-oxoproline and 7-ketodeoxycholic acids were strongly increased in both drug-resistant cell lines (Figure 4A,B, Supplemental Table S1).

**Figure 5:**
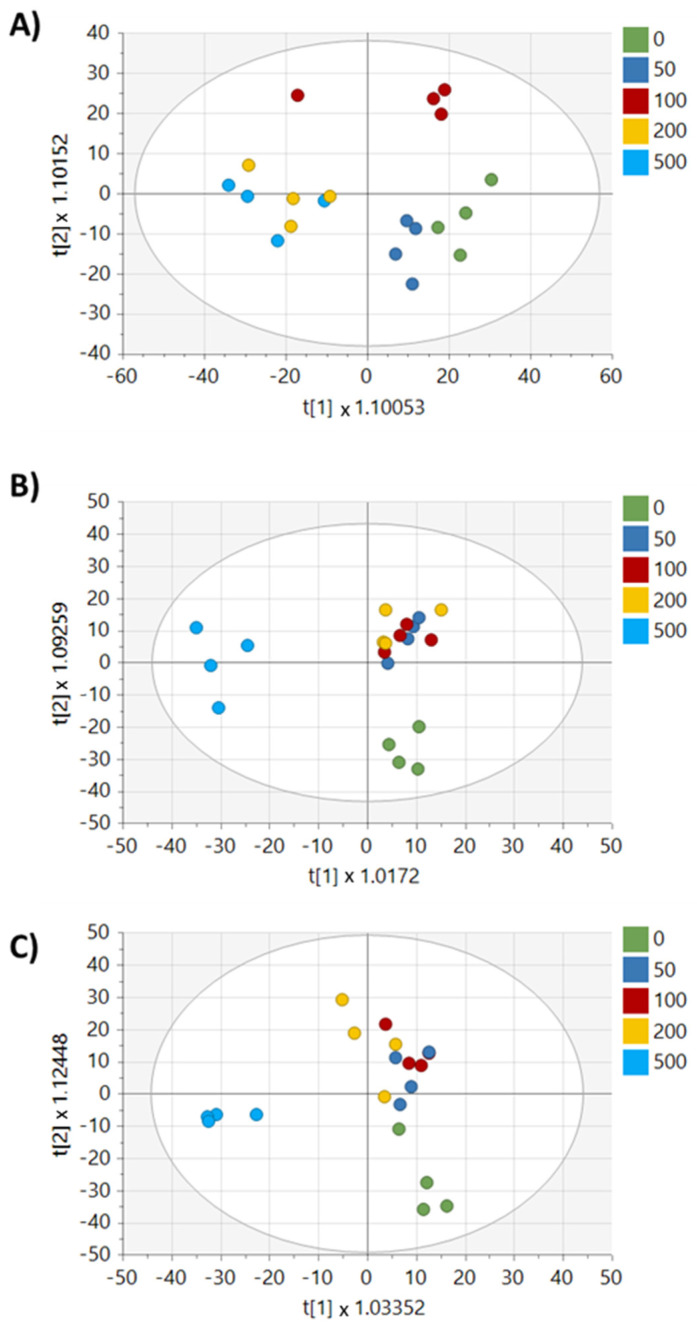
Multivariate analysis of intracellular metabolomics data for each cell line treated across a range of doxorubicin doses. (**A**) OPLS-DA of MDA-MB-231 cells. (**B**) OPLS-DA of DOX-RES-50 cells. (**C**) OPLS-DA of DOX-RES-100 cells. Plots are colored based on doxorubicin treatment concentrations in µM.

To better understand how drug-resistant cells respond metabolically to a repeat exposure, MDA-MB-231, DOX-RES-50, and DOX-RES-100 cells were treated with 0, 50, 100, 200, and 500 µM doxorubicin for 24 h and cells were then analyzed by untargeted metabolomics. Multivariate analysis revealed clear separation between treatment levels, particularly in the lower doses, in MDA-MB-231 cells (Figure 5A). In contrast, DOX-RES-50 and DOX-RES-100 cells showed low separation between lower doxorubicin treatment concentrations, indicating a loss of dose response at lower doxorubicin levels (Figure 5B,C). Correlation analysis was performed using MetaboAnalyst for each cell line to determine in-house matched metabolites that changed in a dose-dependent manner with doxorubicin treatment (Table 1). This analysis revealed 54 metabolites that significantly correlated with doxorubicin in the parental MDA-MB-231 cell line. This number dropped to 17 and 26 for the DOX-RES-50 and DOX-RES-100 cell lines, respectively. Methylthioadenosine, phenylalanine, and N-acetylaspartate were significantly correlated with doxorubicin concentration in all three cell lines. Additionally, glutarate and cytosine were both significantly positively correlated with doxorubicin treatment in the two resistant cell lines, but the correlation was nonsignificant in the parental MDA-MB-231 cell line. Analysis of intracellular doxorubicin signaling in each cell line following each treatment did not show reduced intracellular accumulation of doxorubicin in the resistant cell lines, indicating that the loss of dose response for metabolites was not due to decreased import or increased export of doxorubicin (Supplemental Table S1).

**Figure 6:**
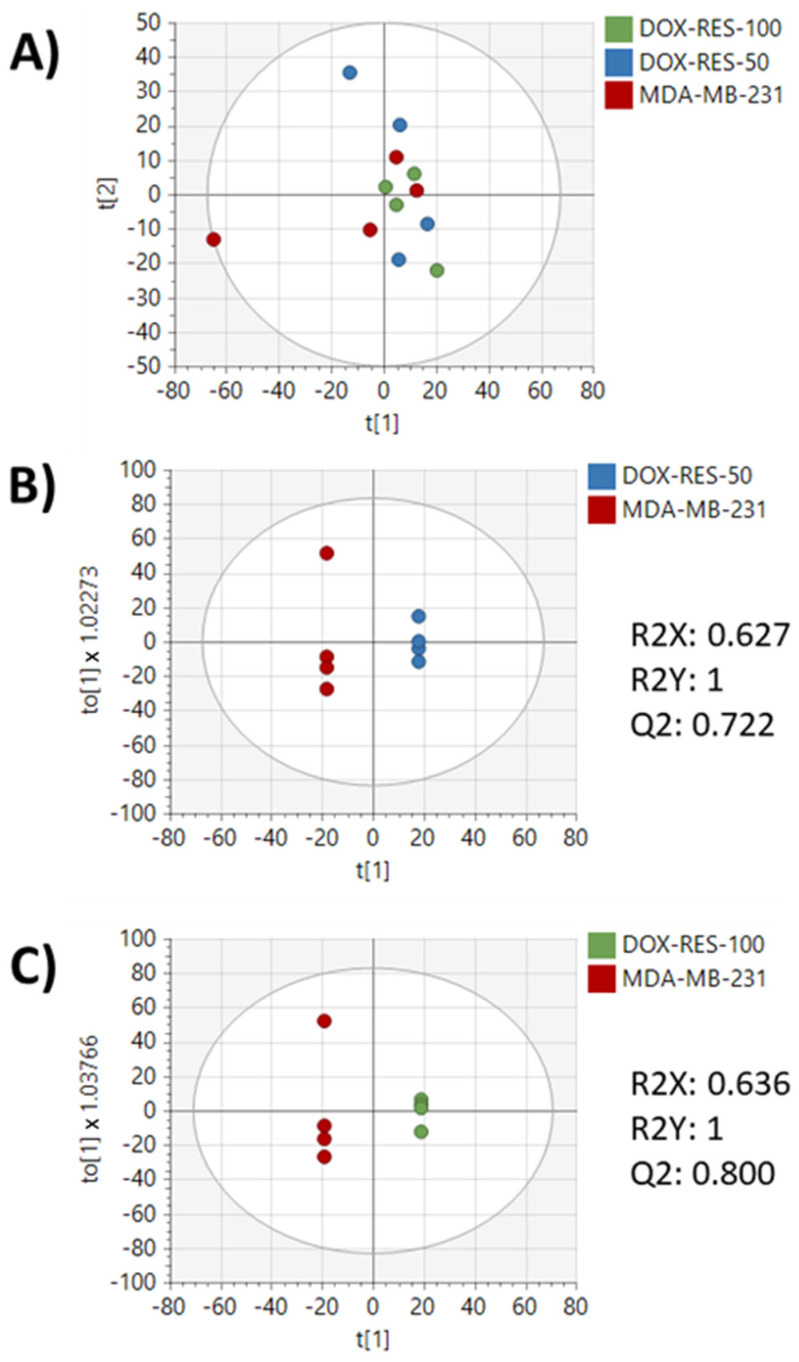
Multivariate analysis of media metabolomics data from each cell line treated with 0 μM doxorubicin. (**A**) PCA of MDA-MB-231, DOX-RES-50, and DOX-RES-100 media samples. (**B**) OPLS-DA of MDA-MB-231 and DOX-RES-50. (**C**) OPLS-DA of MDA-MB-231 and DOX-RES-100.

**Figure 7:**
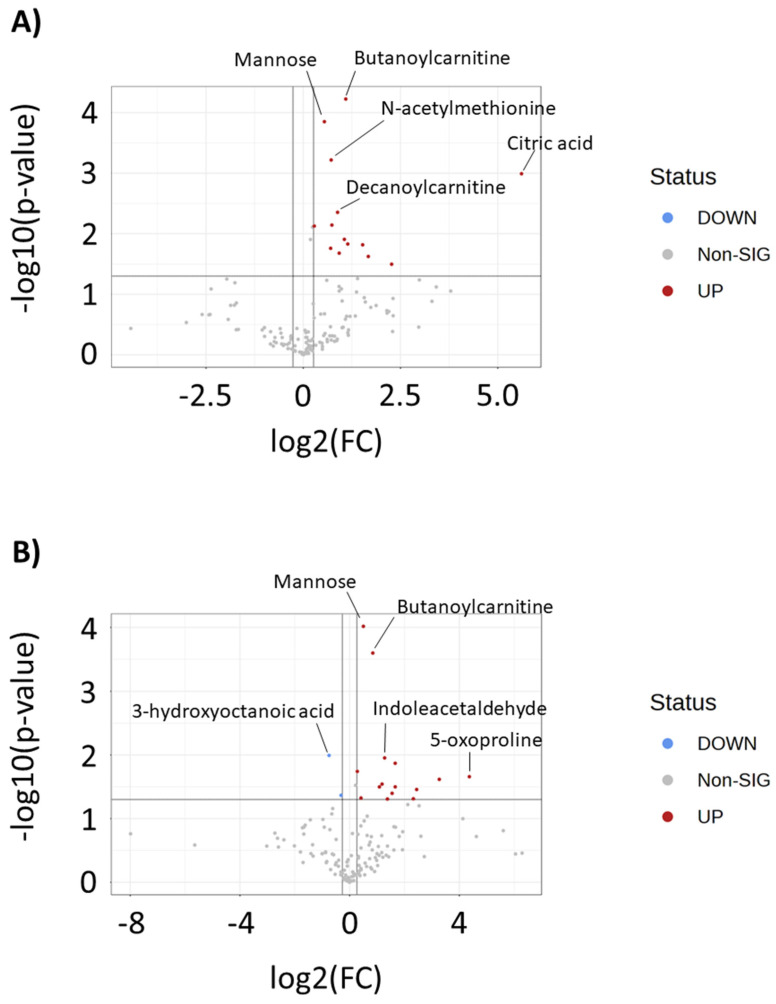
Volcano plots of media in-house matched metabolites that differentiate (**A**) MDA-MB-231 and DOX-RES-50 and (**B**) MDA-MB-231 and DOX-RES-100. Metabolites are highlighted that have a *p* < 0.05 and an absolute fold change ≥ 1.2. Metabolites in red are increased in doxorubicin-resistant cell line samples and metabolites in blue are decreased in doxorubicin-resistant cell line samples.

**Figure 8:**
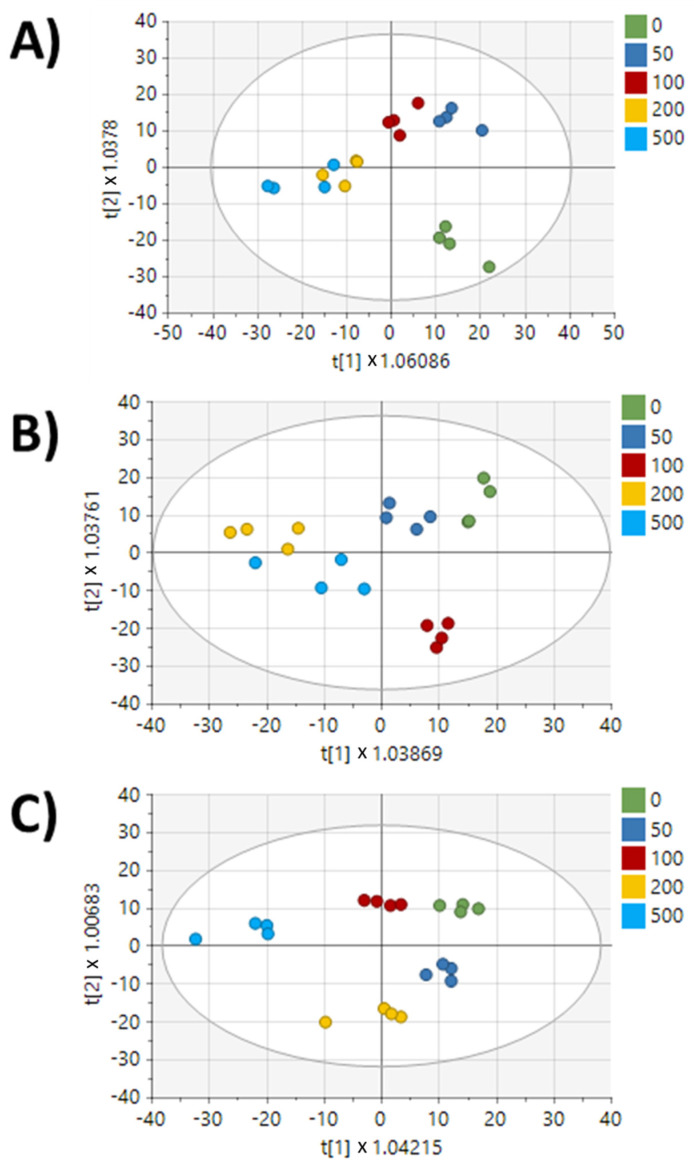
Multivariate analysis of media metabolomics data for each cell line treated across a range of doxorubicin doses. (**A**) OPLS-DA of MDA-MB-231 cells. (**B**) OPLS-DA of DOX-RES-50 cells. (**C**) OPLS-DA of DOX-RES-100 cells. Plots are colored based on doxorubicin treatment concentrations in µM.

Media samples from each cell line were also analyzed by metabolomics to investigate changes in secreted metabolites following resistance acquisition. OPLS-DA, but not PCA, showed differences (R2Y, Q2 > 0.5) in baseline secreted metabolite profiles of doxorubicin-resistant cell lines compared to the parental MDA-MB-231 cell line (Figure 6). Statistical analysis revealed 107 and 133 peaks that were significantly different (*p* < 0.05) in DOX-RES-50 and DOX-RES-100, respectively, compared to the parental MDA-MB-231 cell line (Supplemental Table S2). Volcano plot analysis of in-house matched metabolites revealed that mannose and butanoylcarnitine were the top two (by *p*-value) significantly increased metabolites in both resistant cell lines (Figure 7A,B). Metabolomics analysis was also performed on media samples following repeated exposure to doxorubicin. Multivariate analysis of each cell line at different doxorubicin concentrations showed clearer separation of doses in media samples as compared to intracellular samples (Figure 8). Also, in contrast to intracellular samples, a loss of dose response was not as clearly observed for the media samples. Correlation analysis of media samples following repeated exposure to doxorubicin revealed 20, 11, and 7 in-house matched peaks in MDA-MB-231, DOX-RES-50, and DOX-RES100, respectively, that significantly correlated with doxorubicin treatment concentration (Table 2). Methylthioadenosine, cytosine, and 7-ketodeoxycholic acid were significantly correlated with doxorubicin treatment in all three cell lines.

## 3. Discussion

This study created doxorubicin-resistant cell lines by exposing MDA-MB-231 cells to a single dose of doxorubicin followed by a six-week recovery period. The acquisition of drug resistance was confirmed through an increase in doxorubicin IC_50_ values in both resistant cell lines. While the fold increase in IC_50_ values is more modest compared to other studies, this degree of resistance is more clinically relevant—indeed, other methods of generating drug resistance can lead to fold changes of 100–1000 in IC_50_ levels, but cause cellular changes that are not reflective of this in cancer patients [17]. Our generation of cell lines with modest increases of doxorubicin IC_50_ levels allows for more relevant biological changes to be observed, and may even reflect more accurately the early stages of resistance development. We used an untargeted metabolomics approach to gain insights into metabolic reprogramming during this stage by assessing metabolic differences between drug-resistant and parental MDA-MB-231 cells. Our results showed clear metabolic differences in baseline intracellular and secreted metabolomes of parental and drug-resistant cells. Moreover, we demonstrated that while parental MDA-MB-231 cells had metabolite sets with clear dose-response activity following a dose range of doxorubicin treatment, drug-resistant cells lost dose-response relationships with the majority of these metabolites. Interestingly, a small number of metabolites were significantly correlated with doxorubicin treatment in the resistant cell lines but not the parental MDA-MB-231 cells, suggesting that these metabolites may play a role in suppressing the metabolic changes associated with doxorubicin exposure in resistant TNBC cells.

Pathway analysis (using KEGG maps) of all peaks revealed arginine and proline metabolism, glutathione metabolism, and beta-alanine metabolism as significantly altered in both resistant cell lines. The arginine and proline metabolism pathway connects arginine, proline, and glutamate, as well as their intermediates. This metabolic pathway connects key processes of cancer cell metabolism, including biosynthesis of other amino acids, nucleotide biosynthesis, TCA cycle activity, and polyamine biosynthesis. Interestingly, this pathway is being investigated as a therapeutic target of several cancers through starvation of specific amino acids in this pathway, including arginine, proline, and glutamine [18]. This pathway also directly feeds into glutathione metabolism and beta-alanine metabolism pathways through spermine. The role of glutathione metabolism has been highly studied in the context of cancer drug resistance, including resistance to doxorubicin. Glutathione is well known to play a role in balancing redox levels within the cell, and also conjugates to exogenous agents, including drugs, for clearance. Cancer cells have been observed to increase glutathione levels to increase their buffering capacity towards reactive oxygen species (ROS) levels. Because many chemotherapeutic drugs work by increasing ROS levels, this is a highly effective survival mechanism for cancer cells [19,20]. The connection between beta-alanine, a non-proteinogenic amino acid, and cancer drug response is less clear; however, this metabolite has been shown to modulate glycolytic activity and cellular migration in cervical and renal cancer models [21]. The KEGG map for beta-alanine metabolism also connects with other anabolic pathways, including pyrimidine metabolism and fatty acid biosynthesis, providing a potential role for this pathway in mediating biosynthetic pathways to support enhanced cell growth, which may aid in resisting cytotoxic agents.

Upon investigating metabolites that were matched to our in-house library, we identified several acylcarnitines as significant differentiators of parental MDA-MB-231 cells and the doxorubicin-resistant cell lines. In general, we observed a decrease in short- and medium-chain acylcarnitines and an increase in long-chain acylcarnitines in the doxorubicin-resistant cell lines. Acylcarnitines are intermediates in fatty acid metabolism and are generated during the process of mitochondrial and peroxisomal β-oxidation of fatty acids, making them markers of energy metabolism. Additionally, they have been identified as indicators of metabolic diseases including cardiovascular disease, diabetes, and certain cancers [22]. More recent studies have found a role of the carnitine system in controlling metabolic plasticity, as this pathway is involved in coupling glucose and fatty acid metabolism through the Randle cycle. Excessive activity of fatty acid oxidation (FAO) leads to high levels of NADH and acetyl-CoA, which can promote the conversion of pyruvate into lactate through pyruvate dehydrogenase inhibition, which in turn leads to a myriad of pro-cancer epigenetic and metabolic changes [23]. This makes the carnitine system a potential anticancer target, where the balance between carbohydrate and fatty acid metabolism can be disrupted. In the context of diabetes, increased long-chain acylcarnitines has been shown to be a marker of decoupling between glycolysis and FAO, leading to incomplete breakdown of fatty acids [24,25]. This points towards a potential mechanism where cancer cells decouple glycolysis and FAO during the acquisition of drug resistance, allowing for the rapid and incomplete breakdown of fatty acids to promote other pro-survival pathways. Interestingly, in a recent investigation, we identified that several chemosensitizing dietary compounds—including polyunsaturated fatty acids and polyphenols—significantly increase long-chain acylcarnitines, suggesting that this may also be a mechanism to restore drug response, perhaps if these metabolites are increased past a certain threshold [26]. Additionally, these compounds may also restore glycolysis–FAO coupling mechanisms, increasing the toxic response of the cell to elevated acylcarnitines [27,28].

One of the unique contributions of this study is the analysis of metabolites that reacted in a dose-responsive manner to a range of doxorubicin doses in the drug-resistant cell lines and the parental MDA-MB-231 cell line. The loss of dose response in the metabolome, particularly at lower doxorubicin levels, suggests that the resistant cells rewire cellular metabolism to survive doxorubicin exposure. Our analysis revealed novel metabolic features that are perturbed during resistance acquisition and may represent novel targets that could be targeted to reverse or mitigate doxorubicin resistance in TNBC. Additionally, these metabolites may have potential to be used as markers to be monitored during therapy to guide treatment dose, treatment length, and/or number of treatments. The correlation data also provide additional insights into the mechanism of action of doxorubicin in breast cancer cells, which, despite its widespread clinical use, still remains unclear [29]. Methylthioadenosine (MTA) was significantly negatively correlated with doxorubicin exposure in all cell lines, suggesting a role for this metabolite in mediating response to this drug. MTA has been shown to play a significant role in cancer, controlling polyamine synthesis/methionine salvage pathways as well as regulating apoptosis, invasiveness, and metastasis [30]. Interestingly, MTA accumulation, through the loss of methylthioadenosine phosphorylase (MTAP), has been shown to lead to increased proliferation and resistance to chemotherapeutic drugs, including doxorubicin, in liver cells [31]. While MTA was significantly correlated with doxorubicin exposure in our resistant cell lines, the correlation value was weaker when compared to the parental MDA-MB-231 cell line, suggesting that suppression of MTA response is a potential mechanism of resistance. Additionally, metabolites regulated by MTA, including polyamines and methionine, lost dose dependence in the resistant cell lines, supporting the conclusion that resistant cells decouple MTA from its effector pathways. More studies are needed to identify the role of MTA in the doxorubicin response of breast cancers, including models with more severely resistant cell lines than those used in this study. Additionally, due to the untargeted nature of this study, targeted methods should be performed to confirm the relationship of these metabolites with chemotherapeutic responses.

In conclusion, the findings of this study contribute to our understanding of metabolic reprogramming in drug-resistant breast cancer cells and suggest potential mechanisms and treatment strategies for this challenging clinical problem. Our findings point to a significant role of acylcarnitines/fatty acid oxidation, as well as specific metabolites such as MTA, 5-oxoproline, and 7-ketodeoxycholic acid in the process of acquired drug resistance. Surprisingly, metabolic investigations regarding the acquisition of drug resistance are sparse in the literature, particularly for TNBC. Because of this, our study provides many novel insights into the metabolic changes that occur during TNBC drug resistance, forming the basis for future research to further investigate this process and generate novel findings, such as metabolic targets that could be manipulated to potentially increase drug sensitivity. Further studies are needed to validate the findings of this study and to explore the functional significance of the metabolic changes observed in the drug-resistant cell lines. Additionally, in vivo model systems should also be investigated to determine if these metabolic changes are observed in this setting as well.

## 4. Methods

### 4.1. Chemical Reagents

Optima grade solvents (water with 0.1% formic acid and methanol with 0.1% formic acid) and fetal bovine serum (FBS) were purchased from Fisher Scientific (Waltham, MA, USA). Dubelcco’s Modified Eagle Medium (DMEM) with high glucose and phosphate-buffered saline (PBS) was purchased from Gibco (Grand Island, NY, USA). Doxorubicin was purchased from SelleckChem (Houston, TX, USA). The MDA-MB-231 cell line was purchased from the American Type Culture Collection (ATCC) (Manassas, VA, USA).

### 4.2. Cell Culture and Establishment of Doxorubicin Resistance

MDA-MB-231 cells were cultured according to manufacturer guidelines. Cells were cultured in DMEM supplemented with 10% FBS, 2 mM glutamine, 50 U/mL penicillin, and 50 µg/mL streptomycin. To generate doxorubicin-resistant cells, MDA-MB-231 cells were treated with 50 or 100 nM doxorubicin for 24 h and were allowed to recover in supplemented DMEM for six weeks. To verify resistance, treatment-naïve and treatment-experienced cells were plated into 96-well plates at 2 × 10^4^ cells per well and were allowed to attach overnight. Cells were then treated with an 11-point dose curve of doxorubicin with a top concentration of 10 µM and a dilution series of 1:1 (n = 3–6 per group). Cell viability was assessed using the alamarBlue assay according to manufacturer instructions (Thermo Scientific). GraphPad was used to generate viability dose curves and calculate doxorubicin IC_50_ values. DMSO was used as a vehicle, with a final concentration of 0.1% for all treatments.

### 4.3. Doxorubicin Treatment and Metabolite Extraction

Cells were plated at approximately 80% confluency in 6-well culture plates and were treated with 500, 200, 100, 50, or 0 nM doxorubicin with a final DMSO amount of 0.1% (n = 4 per group). After 24 h of treatment, metabolites were extracted from cell samples as described previously [26,32,33,34]. Briefly, treatment media were aspirated, and cells were washed with 1 mL of ice-cold PBS. After aspirating off PBS, 500 µL of ice-cold 80% methanol was added to culture dishes, and cells were detached using cell scrapers. Protein concentration was assessed by a bicinchoninic acid (BCA) assay and additional 80% methanol was added to each sample to normalize for protein concentration. Samples were vortexed at 5000 rpm for 10 min, centrifuged at 16,000× *g* at 4 °C for 10 min, and supernatants were transferred to autosampler vials for analysis by ultra-high-pressure liquid chromatography–high-resolution mass spectrometry (UHPLC-HRMS). Quality control study pools (QCSP) were created by combining 10 µL of each sample into a single mixture. Method blanks were created by adding 500 µL of 80% methanol to empty tubes and were processed in an identical manner as the study samples.

### 4.4. UHPLC-HRMS Metabolomics Data Acquisition and Preprocessing

Metabolomics data were acquired via previously published UHPLC-HRMS methods [26,32,33,34,35,36,37]. The analysis utilized a Vanquish UHPLC system coupled to a Q Exactive™ HF-X Hybrid Quadrupole-Orbitrap Mass Spectrometer (Thermo Fisher Scientific, San Jose, CA, USA) equipped with an HSS T3 C18 column (2.1 mm × 100 mm, 1.7 µm, Waters Corporation) held at 50 °C. A binary pump was used with water + 0.1% formic acid (A) and methanol + 0.1% formic acid (B) as mobile phases. The mobile phase gradient started from 2% B, increased to 100% B in 16 min, and was then held for 4 min with a flow rate of 400 µL/min. Mass spectral data were collected using a data-dependent acquisition mode in positive polarity at 70–1050 *m*/*z*. QCSP and blank injections were placed at a rate of 10% throughout the study samples. An injection volume of 5 µL was used for analysis of each sample. Raw UHPLC-HRMS data were imported into Progenesis QI (version 2.1, Waters Corporation, MA, USA) for alignment, peak picking, and deconvolution. Background signals were removed by filtering out peaks with a higher average abundance in the blank injections as compared to the QCSP injections. Data were normalized using a QCSP reference sample using the “normalize to all” function in progenesis [38].

### 4.5. Compound Identification/Annotation

Peaks were matched to an in-house library of reference standards or public mass spectral databases from the National Institute of Standards and Technology (NIST) and METLIN. Peaks were matched to metabolites by retention time (RT, ±0.5 min, in-house library only), exact mass (MS, <5 ppm), and fragmentation pattern (MS/MS, similarity score > 30). An ontology system was given to denote the evidence basis for each metabolite assignment. OL1 refers to a match to the in-house library for RT, MS, and MS/MS; OL2a refers to an in-house match to the in-house library for RT and MS; OL2b refers to a match to the in-house library for MS and MS/MS; PDa refers to a match to public databases for MS and MS/MS; PDb refers to a public database match for MS and theoretical MS/MS (HMDB); PDc refers to a public database match for MS and isotopic similarity; PDd refers to a public database match for MS only.

### 4.6. Multivariate, Univariate, and Pathway Analyses

Principal component analysis (PCA) and partial least squares discriminant analysis (OPLS-DA) was performed in SIMCA 16 (Sartorius Stedim Data Analytics AB, Umeå, Sweden) using the normalized, filtered data. Unit Variance (UV) scaling was used for all multivariate plots. PCA plots were used to assess data quality by verifying the clustering and centering of QCSP samples by PCA, and OPLS-DA plots were used to assess the separation of metabolomes between vehicle and treated cells, as well as to calculate variable importance to projection (VIP) scores for each peak. Heatmaps were generated using MetaboAnalyst 5.0 [39]. Fold changes and *p*-values were calculated for each peak for each treatment as compared to the vehicle control. *p*-values were calculated using Student’s *t*-test. Correlation analyses were performed by using the Statistical Analysis (metadata table) module in MetaboAnalyst 5.0 using Pearson r as the correlation measure. *p*-values were not adjusted for multiple testing due to the small sample size of this study and the exploratory, rather than confirmatory, nature of this study [40]. Pathway analyses were conducted using the “Functional Analysis” module of MetaboAnalyst 5.0 using all peaks in the metabolomics dataset. Metabolites were mapped on the KEGG metabolite set library using a *p*-value cutoff of 0.05.

## Figures and Tables

**Table 1:** Intracellular metabolites significantly correlated with doxorubicin treatment across drug-sensitive and drug-resistant cell lines.

Metabolite (Name_Ontology Level_Retention Time_Mass)	MDA-MB-231		DOX-RES-50		DOX-RES-100	
	Correlation	*p*-Value	Correlation	*p*-Value	Correlation	*p*-Value
Methylthioadenosine_OL1_4.92_297.0893 *n*	−0.81	0.00001554	−0.72	0.0003888	−0.68	0.0010676
N-Acetylmethionine_OL1_4.71_191.0610 *n*	−0.77	0.00007602	−0.50	0.023791	−0.33	0.15296
Anthranilate_OL1_5.84_137.0474 *n*	−0.76	0.00009184	0.15	0.53421	−0.27	0.24814
12-Hydroxydodecanoic Acid_OL2b_5.12_261.1439 *m*/*z*	−0.76	0.00011148	−0.30	0.19307	0.29	0.21246
2-Octenedioic Acid_OL2b_1.44_137.0594 *m*/*z*	−0.74	0.00019629	−0.31	0.17985	−0.37	0.11085
Indoleacrylic Acid_OL2b_4.34_187.0632 *n*	−0.74	0.00020622	−0.35	0.13308	−0.50	0.023743
2-Aminocaprylic Acid_OL1_6.37_160.1329 *m*/*z*	−0.72	0.00037329	−0.39	0.088065	−0.49	0.029253
Adenosine 2′,3′-Cyclic Phosphate_OL2b_2.91_330.0592 *m*/*z*	0.71	0.00047735	0.21	0.37223	0.83	0.00000680
Trans-3-Hydroxycinnamate_OL2b_1.56_164.0473 *n*	−0.70	0.00053789	−0.42	0.062772	−0.44	0.051265
Pyridoxine_OL1_1.27_169.0735 *n*	−0.70	0.00054082	0.09	0.70269	0.06	0.79124
Spermidine_OL2b_3.42_146.1649 *m*/*z*	−0.68	0.00099902	−0.14	0.54993	0.16	0.5026
Cytidine_OL1_0.69_243.0848 *n*	0.67	0.0013015	0.18	0.44717	0.16	0.50397
Threonine_OL2a_0.67_158.0211 *m*/*z*	−0.66	0.0015141	−0.47	0.037284	−0.37	0.10919
2-Octenedioic Acid_OL2b_3.72_172.0730 *n*	−0.66	0.0016946	−0.34	0.13976	0.30	0.19228
5-Hydroxyindoleacetate_OL2b_2.91_191.0575 *n*	−0.66	0.001717	−0.28	0.23609	−0.20	0.38949
Indoleacetaldehyde_OL1_4.34_160.0754 *m*/*z*	−0.65	0.0017981	−0.15	0.5315	−0.26	0.27521
N-Acetylaspartate_OL2a_0.83_198.0368 *m*/*z*	0.65	0.0018161	0.27	0.2445	0.43	0.059134
Leucine_OL1_1.70_131.0944 *n*	−0.65	0.0019876	−0.38	0.097942	−0.41	0.071259
Hydroxyphenyllactate_OL1_4.59_205.0468 *m*/*z*	−0.65	0.0020371	−0.23	0.33412	−0.44	0.05153
Indoleacetaldehyde_OL2b_2.46_159.0682 *n*	−0.63	0.0030782	0.06	0.78868	0.06	0.78725
Octanoyl-L-Carnitine_OL1_9.20_288.2162 *m*/*z*	−0.63	0.0031425	−0.42	0.067438	−0.25	0.29713
Isoleucyl-Leucine_OL2b_5.43_245.1852 *m*/*z*	−0.62	0.0035538	−0.66	0.0016847	−0.08	0.73277
L-Tryptophan_OL1_4.34_204.0897 *n*	−0.62	0.0036778	−0.24	0.30013	−0.38	0.1017
L-Phenylalanine_OL1_3.22_165.0787 *n*	−0.61	0.0042322	−0.48	0.033566	−0.54	0.014204
Deoxyadenosine_OL2b_3.57_252.1084 *m*/*z*	−0.60	0.0048732	−0.20	0.39106	−0.29	0.22223
L-Methionine_OL2a_1.07_172.0400 *m*/*z*	−0.59	0.0057758	−0.37	0.11265	−0.25	0.28305
Pantothenate_OL1_4.04_219.1102 *n*	−0.58	0.0068454	0.12	0.60768	0.24	0.31459
Trans-Cinnamic Acid_OL2b_3.24_148.0522 *n*	−0.58	0.0069352	−0.35	0.12974	−0.42	0.063941
Pyridoxal_OL2b_2.81_150.0548 *m*/*z*	−0.58	0.0079745	0.22	0.36202	0.07	0.77067
Leucyl-Leucine_OL1_5.89_245.1852 *m*/*z*	−0.57	0.0083982	−0.34	0.14013	−0.28	0.23375
Indole-3-Aldehyde_OL2b_4.34_146.0599 *m*/*z*	−0.56	0.0094943	−0.24	0.31034	−0.33	0.15284
2-Octenedioic Acid_OL2a_6.83_195.0622 *m*/*z*	0.56	0.010025	−0.17	0.47878	−0.38	0.093781
N-Acetylaspartate_OL1_1.11_175.0478 *n*	0.54	0.013375	0.61	0.0046458	0.50	0.025329
Spermidine_OL2a_0.65_145.1575 *n*	−0.54	0.013553	−0.14	0.56286	0.05	0.82905
Phenylacetylglycine_OL1_5.92_193.0737 *n*	−0.54	0.014787	0.16	0.48874	0.15	0.5273
Dodecenoylcarnitine_OL1_11.80_342.2630 *m*/*z*	−0.53	0.015577	−0.39	0.087782	−0.09	0.70138
Indole-3-Ethanol_OL2b_4.34_144.0806 *m*/*z*	−0.52	0.017664	−0.22	0.34956	−0.26	0.26126
Guanine_OL2b_3.62_152.0563 *m*/*z*	−0.51	0.020217	−0.21	0.36735	−0.18	0.45806
L-Tyrosine_OL2b_3.37_182.0807 *m*/*z*	−0.51	0.020866	−0.09	0.70388	−0.20	0.40599
Threonine_OL1_1.07_102.0548 *m*/*z*	−0.51	0.021699	0.14	0.56117	−0.14	0.56762
Butenylcarnitine_OL2a_2.71_262.1642 *m*/*z*	−0.51	0.022923	0.17	0.47043	0.35	0.126
Creatine_OL2a_0.51_263.1455 *m*/*z*	−0.50	0.024141	0.39	0.093473	0.85	0.00000202
5-Oxoproline_OL2b_0.77_129.0424 *n*	−0.49	0.026792	−0.10	0.68462	−0.04	0.85349
S-Adenosylhomocysteine_OL1_2.09_384.1212 *n*	−0.48	0.030318	0.21	0.38359	−0.07	0.75567
Propionyl-L-Carnitine_OL1_2.09_218.1387 *m*/*z*	−0.48	0.033559	0.34	0.1442	0.15	0.52079
Trigonelline_OL2b_5.41_138.0546 *m*/*z*	0.47	0.035034	−0.35	0.12744	−0.02	0.93339
Creatine_OL2a_0.49_132.0766 *m*/*z*	−0.47	0.036563	0.43	0.055497	0.82	0.00000987
L-Phenylalanine_OL2a_3.22_331.1643 *m*/*z*	−0.47	0.0368	−0.38	0.10306	−0.51	0.022902
N-Acetylhistidine_OL2b_2.24_198.0872 *m*/*z*	−0.46	0.039432	0.28	0.23266	−0.02	0.92921
Spermidine_OL2a_0.49_73.5862 *m*/*z*	−0.46	0.040849	0.10	0.66657	0.22	0.35548
Pantetheine_OL2b_9.30_261.1260 *m*/*z*	0.46	0.041443	−0.08	0.74182	0.18	0.43803
Acetyl-Dl-Carnitine_OL1_1.01_204.1229 *m*/*z*	−0.46	0.041794	−0.16	0.49485	0.13	0.57551
1-Aminocyclopropanecarboxylic Acid_OL2a_0.77_84.0443 *m*/*z*	−0.46	0.043552	−0.02	0.93018	0.00	0.99278
Hexanoylcarnitine_OL1_6.23_260.1850 *m*/*z*	−0.45	0.047391	−0.24	0.2993	0.19	0.4278
4-Acetamidobutanoic Acid_OL2b_3.37_146.0809 *m*/*z*	−0.41	0.075098	0.51	0.020527	0.38	0.099942
Hexanoylcarnitine_OL1_6.53_260.1850 *m*/*z*	−0.40	0.079541	−0.33	0.15004	−0.55	0.011742
Maleic Acid_OL2a_1.11_134.0445 *m*/*z*	0.39	0.087469	0.52	0.017879	0.41	0.074901
Glutamyl-Valine_OL2b_3.72_247.1284 *m*/*z*	−0.39	0.090678	0.39	0.090925	0.53	0.016339
2-Hydroxytetradecanoic Acid_OL2a_14.99_227.1998 *m*/*z*	0.38	0.099925	−0.06	0.81747	0.51	0.021926
Acetyl-DL-Carnitine_OL1_0.71_203.1156 *n*	−0.36	0.11621	0.46	0.039795	0.00	0.99876
Butanoylcarnitine_OL1_3.64_231.1467 *n*	−0.34	0.14514	0.41	0.072478	0.48	0.032961
Glycoursodeoxycholic Acid_OL2b_14.05_449.3136 *n*	−0.32	0.16414	0.47	0.037037	−0.09	0.71422
Sorbitol_OL1_0.65_182.0786 *n*	0.30	0.19459	−0.07	0.78117	0.47	0.037825
Betaine_OL2a_0.67_140.0680 *m*/*z*	−0.30	0.20182	−0.56	0.01107	−0.03	0.90492
Cadaverine_OL2a_0.83_102.1150 *m*/*z*	−0.30	0.20579	0.16	0.49012	0.55	0.011389
2-Methylbutyroylcarnitine_OL1_4.84_246.1696 *m*/*z*	−0.28	0.23419	0.55	0.012568	0.38	0.095206
Tetradecenoyl-L-Carnitine_OL1_12.82_370.2941 *m*/*z*	−0.27	0.24147	−0.61	0.0043994	0.00	0.99629
Creatinine_OL1_0.67_136.0479 *m*/*z*	0.27	0.24167	0.20	0.38662	0.45	0.046976
Citric Acid_OL2a_0.98_176.0083 *m*/*z*	−0.27	0.25513	0.27	0.24076	−0.45	0.048451
Spermine_OL2b_1.97_202.2156 *n*	−0.25	0.27928	0.17	0.48187	0.49	0.028223
7-Ketodeoxycholic Acid_OL2b_15.51_406.2712n	0.25	0.28049	−0.22	0.34463	−0.47	0.038013
Indolelactic Acid_OL1_7.14_205.0735 *n*	−0.25	0.28598	−0.26	0.26977	−0.68	0.00088093
2-Hydroxytetradecanoic Acid_OL2a_15.04_283.1663 *m*/*z*	0.25	0.29295	−0.12	0.60035	0.55	0.012847
Glutarate_OL1_2.66_115.0388 *m*/*z*	−0.24	0.30605	0.52	0.018071	0.55	0.011919
Cytosine_OL1_1.04_112.0503 *m*/*z*	0.22	0.34136	0.83	0.00000607	0.61	0.0043897
B-Nicotinamide Adenine Dinucleotide_OL2a_3.06_333.5691 *m*/*z*	−0.18	0.44516	0.06	0.81435	0.51	0.02153
Cadaverine_OL2a_0.40_102.1150 *m*/*z*	−0.18	0.4511	0.21	0.36841	0.59	0.0061138
Xanthine_OL1_1.54_153.0403 *m*/*z*	−0.15	0.52016	0.69	0.0007609	0.28	0.23164
Creatine_OL1_0.69_131.0693 *n*	0.04	0.8519	0.25	0.27987	0.46	0.039218
Docosatrienoic Acid_OL2a_16.73_299.2728 *m*/*z*	0.00	0.99386	−0.58	0.0073867	0.26	0.27377

Metabolites are named in the following format: metabolite_ontology level_retention time_mass. An “*m*/*z*” following the mass denotes an ion mass, whereas an “*n*” denotes a neutral mass. The names given for each match are based on the names of the reference standards run on our UHPLC-HRMS platform or the names provided in public databases. This method does not necessarily differentiate between some isomeric forms, such as D and L enantiomers. Positive correlation values indicate metabolites that positively correlate with doxorubicin treatment.

**Table 2:** Media metabolites significantly correlated with doxorubicin treatment across drug-sensitive and drug-resistant cell lines.

Metabolite (Name_Ontology Level_Retention Time_Mass)	MDA-MB-231		DOX-RES-50		DOX-RES-100	
	Correlation	*p*-Value	Correlation	*p*-Value	Correlation	*p*-Value
Cytosine_OL1_1.04_112.0503 *m*/*z*	0.84937	0.00000217	0.59474	0.0056768	0.67691	0.001046
Methylthioadenosine_OL1_4.92_297.0893 *n*	−0.79527	0.0000278	−0.87672	0.000000396	−0.79282	3.06 × 10^−5^
Malic Acid_OL2a_0.86_157.0103 *m*/*z*	0.58391	0.0068684	0.0039314	0.98688	−0.020673	0.93106
Threonine_OL1_1.07_102.0548 *m*/*z*	0.56734	0.009083	0.29776	0.2023	−0.040839	0.86426
Butanoylcarnitine_OL1_3.64_231.1467 *n*	0.56564	0.0093398	0.015986	0.94667	0.16808	0.47874
Xanthine_OL1_1.54_153.0403 *m*/*z*	0.55436	0.011196	0.43665	0.054237	0.41854	0.066265
N-Methyl-D-Aspartic Acid_OL2a_0.65_147.0526 *n*	−0.54143	0.013681	−0.25443	0.27903	0.39605	0.083868
Glutarate_OL2b_4.07_115.0388 *m*/*z*	0.53065	0.016078	0.32205	0.16614	−0.20547	0.38482
Indoleacetaldehyde_OL1_4.34_160.0754 *m*/*z*	0.52999	0.016234	0.094866	0.69075	0.10551	0.65797
3-(Carbamoylamino)Propanoic Acid_OL2a_1.07_177.0240 *m*/*z*	−0.52048	0.018636	0.4759	0.033922	−0.045774	0.84803
N-Acetylserine_OL2a_1.16_148.0600 *m*/*z*	−0.51527	0.020066	−0.1719	0.46865	0.0052969	0.98232
12-Hydroxydodecanoic Acid_OL2b_5.12_261.1439 *m*/*z*	0.51409	0.020401	−0.23781	0.31268	−0.064887	0.78579
Indoleacetaldehyde_OL2b_2.46_159.0682 *n*	0.50304	0.02377	−0.21828	0.35521	−0.22613	0.33773
Nicotinamide_OL1_1.23_123.0551 *m*/*z*	0.48868	0.028787	0.10396	0.66271	−0.20153	0.39419
Fructose_OL2a_0.88_113.0206 *m*/*z*	−0.48047	0.03201	−0.22229	0.34621	−0.037041	0.87679
L-Phenylalanine_OL2a_3.22_331.1643 *m*/*z*	0.47757	0.033214	0.32444	0.16283	0.28265	0.22724
Pantothenate_OL1_4.04_219.1102 *n*	0.46838	0.037262	0.082628	0.7291	0.0097354	0.96751
7-Ketodeoxycholic Acid_OL2b_15.51_406.2712 *n*	−0.45131	0.045786	−0.59553	0.0055969	−0.61972	0.003564
Glutamyl-Valine_OL2b_3.72_247.1284 *m*/*z*	0.44907	0.047004	0.061125	0.79795	−0.076558	0.74836
1-Methyl-L-Histidine_OL2a_0.58_192.0737 *m*/*z*	0.44537	0.049076	−0.25272	0.28237	0.10562	0.65765
Creatinine_OL1_0.65_114.0661 *m*/*z*	0.39497	0.084797	0.051977	0.82772	−0.47478	0.034403
Sphinganine_OL2b_17.24_284.2941 *m*/*z*	0.38174	0.096742	0.44605	0.048695	0.14411	0.54441
Guanine_OL2b_3.62_152.0563 *m*/*z*	−0.29382	0.20862	−0.49466	0.026606	−0.37563	0.10266
Prostaglandin B2_OL2b_15.92_357.2030 *m*/*z*	0.2713	0.24726	−0.44964	0.046693	0.18064	0.44597
Histidine_OL1_0.58_155.0693 *n*	−0.18863	0.42576	0.61221	0.0041156	0.20709	0.381
Arginine_OL1_0.58_175.1188 *m*/*z*	−0.14538	0.54083	0.58944	0.0062371	0.40546	0.076132
Deoxyadenosine_OL2b_3.57_252.1084 *m*/*z*	−0.087792	0.71284	−0.44602	0.048709	−0.32382	0.16369
L-Carnitine_OL2a_0.69_144.1017 *m*/*z*	0.080419	0.73609	0.016996	0.9433	0.47174	0.035739
Lysine_OL1_0.51_146.1053 *n*	−0.079963	0.73754	0.40311	0.078011	0.56567	0.009334
2-Hydroxypyridine_OL2b_1.23_96.0443 *m*/*z*	−0.034015	0.88679	0.56715	0.0091119	0.28764	0.21879
Pipecolate_OL1_0.51_130.0861 *m*/*z*	−0.0041201	0.98625	0.42884	0.059206	0.51643	0.019741

## Data Availability

The data presented in this study are available in article and supplementary material.

## Supplementary Materials

The following supporting information can be downloaded at: https://www.mdpi.com/article/10.3390/metabo13070865/s1, Table S1: Peak information and statistics for intracellular metabolomics samples; Table S2: Peak information and statistics for extracellular metabolomics samples.
